# Retraction: Colloid Cyst: A Potentially Life-Threatening Etiology of Severe Headache in a Patient With Migraine

**DOI:** 10.7759/cureus.r84

**Published:** 2024-01-25

**Authors:** Rayan N Alshuaylan, Abdulelah A Alismail, Fisal M Haobani, Mohammed R Alfulayw, Abdullah Y Abu Maghayed, Alya A Khashoggi, Shahid A Al-Mahdi, Sarah A AlKishi, Jawad H Alnaqaa, Shahad B Alwazzan, Malak Alshammari

**Affiliations:** 1 College of Medicine, Shaqra University, Shaqra, SAU; 2 College of Medicine, King Faisal University, Al-Ahsa, SAU; 3 College of Medicine, Vision Colleges, Riyadh, SAU; 4 College of Medicine, Imam Abdulrahman Bin Faisal University, Dammam, SAU; 5 College of Medicine, Majmaah University, Al-Majma'ah, SAU; 6 College of Medicine, King Saud Bin Abdulaziz University for Health Sciences, Riyadh, SAU; 7 College of Medicine, Albaha University, Abha, SAU; 8 General Surgery, Al Kharj Military Industries Corporation Hospital, Al-Kharj, SAU

The Editors-in-Chief have retracted this article. Concerns were raised regarding the identity of the authors on this article. Specifically, Faisal Alhaway and Malak Shammari have stated that they were added to this article without their knowledge or approval. The identity of the other authors could also not be verified. As the appropriate authorship of this work cannot be established, the Editors-in-Chief no longer have confidence in the results and conclusions of this article.

